# Humidity – The overlooked variable in the thermal biology of mosquito-borne disease

**DOI:** 10.1111/ele.14228

**Published:** 2023-05-10

**Authors:** Joel J. Brown, Mercedes Pascual, Michael C. Wimberly, Leah R. Johnson, Courtney C. Murdock

**Affiliations:** 1 Department of Entomology, Cornell University, Ithaca, New York, USA; 2 Department of Ecology and Evolution, University of Chicago, Chicago, Illinois, USA; 3 Department of Geography and Environmental Sustainability, University of Oklahoma, Norman, Oklahoma, USA; 4 Department of Statistics, Virginia Polytechnic Institute and State University, Blacksburg, Virginia, USA

**Keywords:** climate change, ecophysiology, humidity, mosquito, parasite ecology, pathogen, temperature, vector-borne disease

## Abstract

Vector-borne diseases cause significant financial and human loss, with billions of dollars spent on control. Arthropod vectors experience a complex suite of environmental factors that affect fitness, population growth and species interactions across multiple spatial and temporal scales. Temperature and water availability are two of the most important abiotic variables influencing their distributions and abundances. While extensive research on temperature exists, the influence of humidity on vector and pathogen parameters affecting disease dynamics are less understood. Humidity is often underemphasized, and when considered, is often treated as independent of temperature even though desiccation likely contributes to declines in trait performance at warmer temperatures. This Perspectives explores how humidity shapes the thermal performance of mosquito-borne pathogen transmission. We summarize what is known about its effects and propose a conceptual model for how temperature and humidity interact to shape the range of temperatures across which mosquitoes persist and achieve high transmission potential. We discuss how failing to account for these interactions hinders efforts to forecast transmission dynamics and respond to epidemics of mosquito-borne infections. We outline future research areas that will ground the effects of humidity on the thermal biology of pathogen transmission in a theoretical and empirical framework to improve spatial and temporal prediction of vector-borne pathogen transmission.

## INTRODUCTION

Vector-borne parasites are common, important biological enemies of humans, animals and plants, transmitted by one living organism to another. Despite the recent gains in reducing the overall global burden for parasites like malaria (Ashepet et al., 2021; Bhatt et al., 2015; Gething et al., 2010), vector-borne diseases still account for 17% of all infectious diseases and cause 700,000 deaths in humans annually (WHO, 2020). Livestock and crop systems are also plagued by vector-borne diseases, which place serious constraints on agricultural production globally (Döring, 2017; Garros et al., 2017), and vector-borne diseases can be devastating in wildlife populations, particularly when introduced to new areas. Collectively, tens of billions of dollars are spent every year on control, medical interventions and mitigating loss of productivity (Aguirre, 2017; George et al., 2015; Stuchin et al., 2016; Warner, 1968; Weaver et al., 2018).

The dependence of many pathogens on ectothermic arthropod vectors for transmission means that vector-borne diseases are highly sensitive to variation in the environment. Arthropod vectors experience a complex suite of environmental factors, both abiotic (e.g., temperature, rainfall, humidity, salinity) and biotic (e.g., biological enemies, inter- and intraspecific interactions and variation in habitat quality). These factors vary in their relative effects on organismal fitness, can synergize (Huxley et al., 2021, 2022; Kleynhans & Terblanche, 2011; Liu & Gaines, 2022), and exert their effects at different spatial scales (Cohen et al., 2016) with important consequences for the abundance and distribution of arthropod vectors (Evans et al., 2019; Ryan et al., 2015), vector population dynamics (Murdock et al., 2017) and pathogen transmission (Huber et al., 2018; Mordecai et al., 2013; Mordecai et al., 2017; Murdock et al., 2016; Murdock, Blanford, Hughes, et al., 2014a; Ngonghala et al., 2021; Samuel et al., 2011; Shocket, Vergara, et al., 2018b; Tesla et al., 2018; Wimberly et al., 2020).

In vector ecology, there has been a strong emphasis on studying the effects of temperature on mosquito-borne pathogen transmission (reviewed in Mordecai et al., 2019). In addition to temperature, water availability is another critical abiotic variable influencing ectotherm biology, and both play important roles determining the distribution and abundance of ectotherms (Chown & Nicolson, 2004; Deutsch et al., 2008; González-Tokman et al., 2020; Kearney & Porter, 2009; Lenhart et al., 2015; Roura-Pascual et al., 2011; Rozen-Rechels et al., 2019; Steiner et al., 2008; van Klink et al., 2020) and species richness (Beck et al., 2017; Calatayud et al., 2016; Cardoso et al., 2020; Hamann et al., 2021; Jamieson et al., 2012; Pilotto et al., 2020). Body temperature has important effects on the rates of enzymatic processes as well as the structural integrity of cellular membranes and proteins (Angilletta, 2009), while all cellular processes rely on water as a solvent for biochemical reactions and for trafficking nutrients into, within, and out of cells (Chaplin, 2006; Chown & Nicolson, 2004). Temperature also affects the amount of desiccation stress an organism experiences due to the fundamental relationship between ambient temperature and the amount of water the surrounding air can hold (Lawrence, 2005; Romps, 2021). Other fields at the climate-health interface have explored the effects of wet heat vs dry heat on the energy budgets of endotherms in the context of human heat stress and climate change (Buzan & Huber, 2020). We anticipate that variation in relative humidity is also an important force shaping the thermal performance of ectotherms, including mosquitoes. Whereas metabolic theory has been well developed and widely applied in ecology to understand temperature effects (Brown et al., 2004; Corkrey et al., 2016; Dell et al., 2011) we currently lack a similar framework for understanding how humidity and temperature interact to influence mosquitoes and their pathogens.

In this Perspectives, we explore the effects of humidity on the thermal performance of mosquito-borne pathogen transmission. We begin by summarizing what is currently known about how temperature and humidity affects mosquito fitness, population dynamics and pathogen transmission, whilst highlighting current knowledge gaps. We present a conceptual framework for understanding the interaction between temperature and humidity and how it shapes the range of temperatures across which mosquitoes persist and achieve high transmission potential. We then discuss how failing to account for these interactions across climate variables hinders efforts to forecast transmission dynamics and to respond to epidemics of mosquito-borne infections. We end by outlining future research areas that will ground the effects of humidity on thermal performance of pathogen transmission in a theoretical and empirical basis to improve spatial and temporal prediction of vector-borne pathogen transmission. Such a framework will inform multiple fields (thermal, disease and landscape ecology and epidemiology) and a diversity of vector-borne disease systems (human, wildlife, domestic animals and plants).

## THE EFFECTS OF TEMPERATURE ON MOSQUITO POPULATION DYNAMICS AND PATHOGEN TRANSMISSION

**FIGURE 1:**
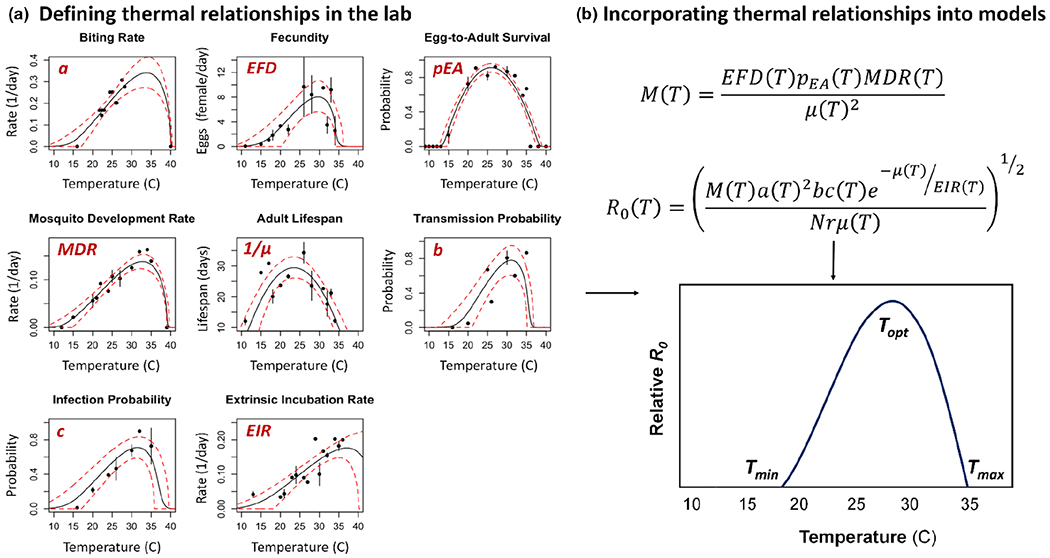
(a) Similar to other ectothermic organisms, the life history traits of mosquitoes and the pathogens they transmit typically exhibit non-linear relationships with environmental temperature, where trait performance is constrained by both cool and warm temperatures and optimized at some intermediate temperature. Further, the effect of temperature on these individual traits can vary qualitatively and quantitatively, resulting in different temperature ranges across which trait performance can occur, temperatures that maximize trait performance, and the overall shape of the temperature-trait relationship (e.g., symmetric vs. asymmetric). As a result, predicting the effects of temperature on mosquito fitness, population growth rates or pathogen transmission is complex. (b) Mathematical models of vector-borne pathogen transmission that incorporate these temperature-trait relationships generally predict transmission to also follow a non-linear relationship and to peak at some intermediate temperature, as depicted here with the temperature-dependent relative reproductive number *R*_*0*_ as a conceptual example. This model incorporates the effects of temperature on traits that drive mosquito population dynamics (e.g., per capita mosquito development rate (*MDR*), the probability of egg to adult survival (*pEA*) and the per capita number of eggs females produce per day (*EFD*)), host-vector contact rates (the per capita daily biting rate of female mosquitoes (*a*)) and the number of mosquitoes alive and infectious (transmission (*b*) and infection (*c*) probabilities, the extrinsic incubation period (1/*EIR*) and the per capita mosquito mortality rate (*μ*)). Where the predicted thermal minimum (*T*_min_), maximum (*T*_max_) and optimum (*T*_opt_) for transmission occur will be dependent upon the relative effect of each trait, the nature of the temperature-trait relationship, and how these factors combine to shape the transmission process. Adapted from Mordecai et al. (2017).

Numerous studies have demonstrated that mosquito-borne pathogen transmission is both seasonally and geographically limited at various spatial scales by variation in ambient temperature (e.g., malaria (Ryan et al., 2015; Siraj et al., 2014; Villena et al., 2022), Zika (Ryan, Carlson, et al., 2020a; Siraj et al., 2018; Tesla et al., 2018), chikungunya (Johansson et al., 2014) and dengue (Mordecai et al., 2017)). The effects of temperature on ectotherm performance, including mosquito vectors, are typically non-linear, with performance steadily increasing from zero at a minimum critical temperature (CT_min_) up to an optimum temperature (*T*_opt_), followed by a steep decline towards the critical thermal maximum (CT_max_) (Figure 1). The CT_min_ and CT_max_ represent the operational limits for trait performance because temperatures that exceed their range are not permissive for ectotherm development, survival or reproduction (Brown et al., 2004; Corkrey et al., 2016; Deutsch et al., 2008; Hoffmann et al., 2013; Sinclair et al., 2016). These thermal limits in ectotherm performance are consistent with the metabolic theory of ecology, which posits that organismal physiological and enzymatic rates will increase predictably with temperature because of increased efficiency of biochemical reactions (Huey & Kingsolver, 2019) up to *T*_opt_. The steep decline in performance above the *T*_opt_ is attributed to the declining efficiency of metabolic processes due to decreases in protein stability as temperatures increase, eventually resulting in organismal death at the *T*_max_. Collectively, this information gives us a Thermal Performance Curve (TPC), which can be used to infer ecological and evolutionary outcomes.

Mosquitoes, like other ectotherms, are highly susceptible to changes in ambient temperature, which demonstrably affects their growth rate (Alto & Juliano, 2001; Delatte et al., 2009; Evans, Newberry, & Murdock, 2018a; Huxley et al., 2022; Monteiro et al., 2007; Paaijmans et al., 2013; Tun-Lin et al., 2000), reproduction (Carrington et al., 2013; Miazgowicz et al., 2020), metabolic rate (Vorhees et al., 2013), lifespan (e.g., Alto & Juliano, 2001; Christofferson & Mores, 2016; Gunay et al., 2010; Miazgowicz et al., 2020), biting rate (Afrane et al., 2005; Lardeux et al., 2008; Miazgowicz et al., 2020; Shapiro et al., 2017), immunity (Adelman et al., 2013; Ferreira et al., 2020; Murdock et al., 2012, 2013; Murdock, Blanford, Luckhart, & Thomas, 2014b; Suwanchaichinda & Paskewitz, 1998), and ability to acquire, carry and transmit pathogens (Johnson et al., 2015; Lambrechts et al., 2011; Mordecai et al., 2013, 2017; Murdock et al., 2016; Murdock, Blanford, Luckhart, & Thomas, 2014b; Paaijmans et al., 2012; Shocket et al., 2020; Shocket, Ryan, & Mordecai, 2018a; Tesla et al., 2018) in a non-linear, unimodal fashion. These temperature-trait relationships can vary in overall shape (e.g., symmetric or asymmetric non-linear relationships) due to differences in the temperatures that optimize and constrain various traits, which in combination will determine the predicted thermal minimum, maximum and optimum for mosquito fitness, intrinsic growth rates of mosquito populations, and pathogen transmission (Figure 1).

Process-based models, which traditionally have relied upon temperature relationships grounded in metabolic theory, have enhanced our ability to predict the effects of environmental drivers on spatial and temporal dynamics of vector-borne disease. Several key biological insights have resulted from this general approach. First, temperate areas of the world that currently experience relatively cool temperatures are expected to increase in thermal suitability for many mosquito-borne diseases with future climate warming (Ryan et al., 2015; Ryan, Carlson, et al., 2020a; Siraj et al., 2014; Tesla et al., 2018), and, in temperate regions, mosquito-borne pathogens can invade or spread during the summer in seasonally varying environments (Huber et al., 2018; Ngonghala et al., 2021). Secondly, areas that are currently permissive (near the *T*_opt_) or warmer than the *T*_opt_ for transmission are expected to experience a decline in thermal suitability with future warming (Murdock et al., 2016; Ryan et al., 2015; Ryan, Lippi, & Zermoglio, 2020b). Third, because mosquito and pathogen species can have different qualitative and quantitative relationships with temperature (resulting in different CT_min_, CT_max_ and *T*_opt_) (Johnson et al., 2015; Miazgowicz et al., 2020; Mordecai et al., 2013, 2017, 2019; Shapiro et al., 2017; Shocket et al., 2020; Shocket, Ryan, & Mordecai, 2018a; Tesla et al., 2018; Villena et al., 2022), shifts in thermal suitability with climate and land-use change could also alter the prevalence and magnitude of mosquito-borne diseases in a given area (Tesla et al., 2018), such as sub-Saharan Africa (Mordecai et al., 2020). Fourth, small variations in ambient temperature at fine-spatial scales can contribute to high heterogeneity in predicted suitability for pathogen transmission across various environments (Afrane et al., 2005; Cator et al., 2013; Evans et al., 2019; Murdock et al., 2017; Okech et al., 2004; Paaijmans & Thomas, 2011; Pincebourde et al., 2016; Thomas et al., 2018; Verhulst et al., 2020; Wimberly et al., 2020), which can have important ramifications for predicting mosquito-borne pathogen transmission and targeting interventions (Thomas et al., 2018; Wimberly et al., 2020). Finally, disease intervention efforts can also be directly or indirectly affected by variation in ambient temperature. Various insecticides (Akinwande et al., 2021; Glunt et al., 2014), entomopathogenic fungi (Darbro et al., 2011; Kikankie et al., 2010) and *Wolbachia* transinfections (Foo et al., 2019; Gu et al., 2022; Murdock, Blanford, Hughes, et al., 2014a; Ross et al., 2017, 2019, 2020; Ulrich et al., 2016) are thermally sensitive, indicating that the efficacy and cost of these interventions could vary seasonally, across geographic regions and with future climate and land-use change (Parham & Hughes, 2015).

## THE EFFECTS OF HUMIDITY ON MOSQUITO FITNESS, POPULATION DYNAMICS AND PATHOGEN TRANSMISSION

Spatial and temporal variation in atmospheric moisture has important implications for an organism’s ability to hydroregulate (Box 1). Hydroregulation is defined as the suite of physiological and behavioural responses organisms utilize to regulate water balance and tolerate dehydrating environmental conditions (Benoit, 2010; Chown et al., 2011; Chown & Nicolson, 2004; Edney, 2012; Lucio et al., 2013). The relationship between organismal fitness and optimal hydroregulation is complex, with significant costs to fitness (e.g., decreased survival and reproduction) occurring when organisms become dehydrated (Anderson & Andrade, 2017; Mitchell & Bergmann, 2016) or overhydrated (Chown & Nicolson, 2004). Insects have a suite of adaptations to conserve water, like physiological changes in skin or cuticular permeability (Rajpurohit et al., 2008; Wu & Wright, 2015), differential regulation of urine and faeces production (Durant et al., 2021; Durant & Donini, 2019; Lajevardi et al., 2021; Weihrauch et al., 2012), and behavioural changes in activity (Kühnholz & Seeley, 1997; Ostwald et al., 2016). Insects also can mitigate water loss by regulating water intake via changes in water utilization, food sources and selection of specific habitats (Benoit, 2010; Bezerra Da Silva et al., 2019; Hagan et al., 2018)). Finally, insects can also produce water via metabolic processes (Chown et al., 2011; Jindra & Sehnal, 1990). Maintaining water balance is a particular challenge for blood-feeding (haematophagic) vectors (Chappuis et al., 2013; Kleynhans & Terblanche, 2011), like mosquitoes (Edney, 2012), where the act of taking a blood meal results in overhydration that requires specialized adaptations for the excretion of water, which, in turn, enhances susceptibility to desiccation overall (Benoit & Denlinger, 2010).

Instead of measuring humidity directly (Box 1), many studies use related variables, like seasonal precipitation or land cover to predict mosquito population dynamics or pathogen transmission (Abdelrazec & Gumel, 2017; Chandy et al., 2013; Chaves & Kitron, 2011; Johansson et al., 2009; Nosrat et al., 2021; Sang et al., 2017; Soti et al., 2012). Mosquito-borne diseases generally peak during, or following, periods of highest rainfall (Chowdhury et al., 2018; Karim et al., 2012; Magombedze et al., 2018; McLaughlin et al., 2019). Rainfall can matter as a standalone variable, since standing water is essential for mosquitoes’ ontogenetic development. However, the effect of precipitation on mosquito population dynamics and disease transmission can operate through other factors that covary with precipitation, such as increased humidity and shifts in temperature that impact mosquito development rates, adult survival and reproduction, parasite development rates and mosquito-human contact rates. The relationship between mosquitoes and precipitation is even more difficult to discern for mosquito species that develop in artificial, human watered containers, where complex interactions can occur between amount of rainfall and access to piped water (Brown et al., 2014; Hayden et al., 2010; Lippi et al., 2018; Padmanabha et al., 2010; Schmidt et al., 2011; Stewart Ibarra et al., 2013). Similarly, measures of land cover such as the normalized difference vegetation index (NDVI) have been used to account for areas too dry for widespread mosquito habitat (Ryan et al., 2015). Ultimately, the use of these proxy measures obscures our understanding of how relative humidity and other environmental variables affect transmission, which, in turn, constrains our ability to predict how mosquito-borne pathogens will respond to future climate and land-use change.

**FIGURE 2:**
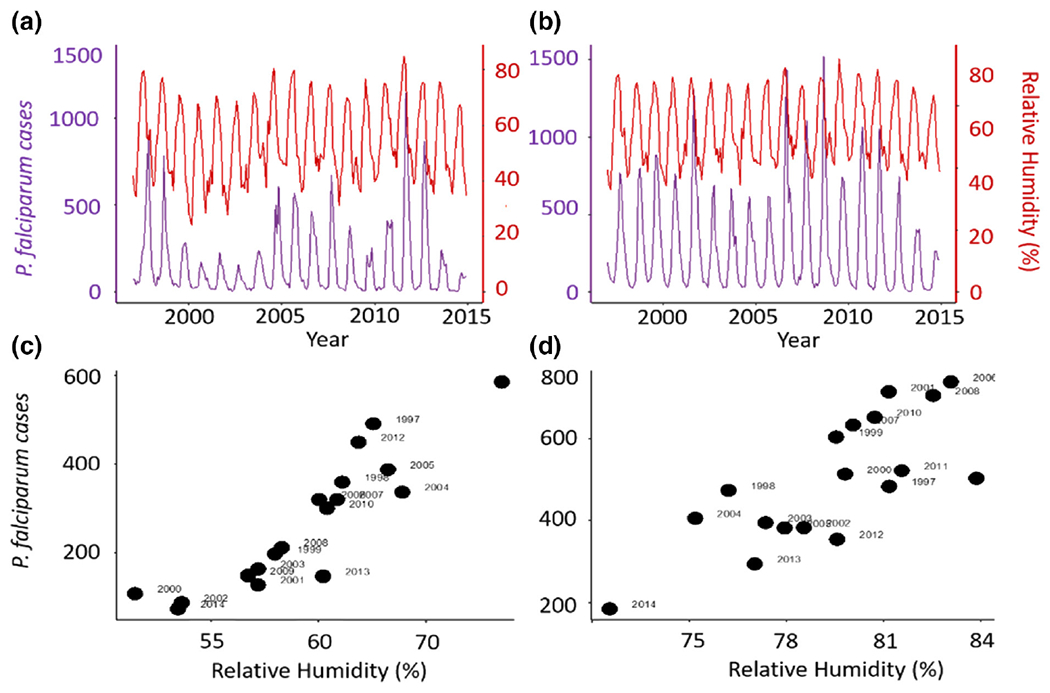
Monthly malaria case data for *Plasmodium falciparum* shown (in purple) with a corresponding time series for relative humidity (RH, red) for two cities in India, Ahmedabad (a) and Surat (b). Total cases during the transmission season from August to November are shown as a function of mean RH in a critical time window preceding this season and including the monsoons from May to July for Ahmedabad (c) and March to July for Surat (d). Figure is taken from Santos-Vega et al. (2022) *Nature Communications* doi: 10.1038/s41467-022-28,145-7. Figure is reproduced under Creative Commons Attribution 4.0 International Licence.

Several studies have demonstrated statistical associations between humidity and mosquito abundance, as well as vector-borne disease incidence and prevalence (Althouse et al., 2015; Asigau & Parker, 2018; Azil et al., 2010; Buckner et al., 2011; Chen et al., 2010; Davis et al., 2018; Diallo et al., 2019; Evans et al., 2019; Jemal & Al-Thukair, 2018; Karim et al., 2012; Lega et al., 2017; Mayne, 1930; Santos-Vega et al., 2022). For example, the sizes of seasonal malaria epidemics in two cities in India exhibit a clear association with relative humidity (Figure 2), with a higher correlation than for temperature or rainfall (Santos-Vega et al., 2016). A semi-mechanistic epidemiological model that incorporates this effect of relative humidity on the transmission rate parameter accurately predicts the temporal dynamics of the disease, including the multiyear cycles in the size of seasonal epidemics (Santos-Vega et al., 2016, 2022). Such predictions can inform mosquito control efforts and targeting prophylaxes. However, the underlying biology of the relationships that exist between humidity and these response variables are often assumed and based on a limited number of empirical studies (summarized in Table 1). Experimental work has thus far shown generally positive effects of increased relative humidity on mosquito survival and desiccation tolerance, production and development of eggs and mosquito activity (up to 90% relative humidity). In contrast, biting rates exhibited increases when conditions are drier and the effect of humidity on vector competence is less clear (Table 1).

Despite the existing body of research, we still do not have a sufficient knowledge base for incorporating the effects of humidity into the current temperature-trait modelling framework. Results from observations studies cannot necessarily be extrapolated to new locations or into the future. Further, the effects of humidity on mosquito and pathogen fitness described by experimental / causation studies are of limited resolution, typically exploring a limited number of humidity levels and encompassing only a handful of mosquito species. The need to better incorporate humidity effects is not unique to vector-borne diseases, but parallels trends seen in the larger body of ecological work on the effects of climate variability and climate change on heat health in ectotherms (Gunderson & Stillman, 2015; van Heerwaarden & Sgrò, 2014). In the following section, we outline how variation in relative humidity interacts with temperature to change the thermal performance of ectothermic vectors and, consequently, pathogen transmission.

## CONSIDERING THE COMBINED EFFECTS OF TEMPERATURE AND HUMIDITY ON TRANSMISSION

**FIGURE 3:**
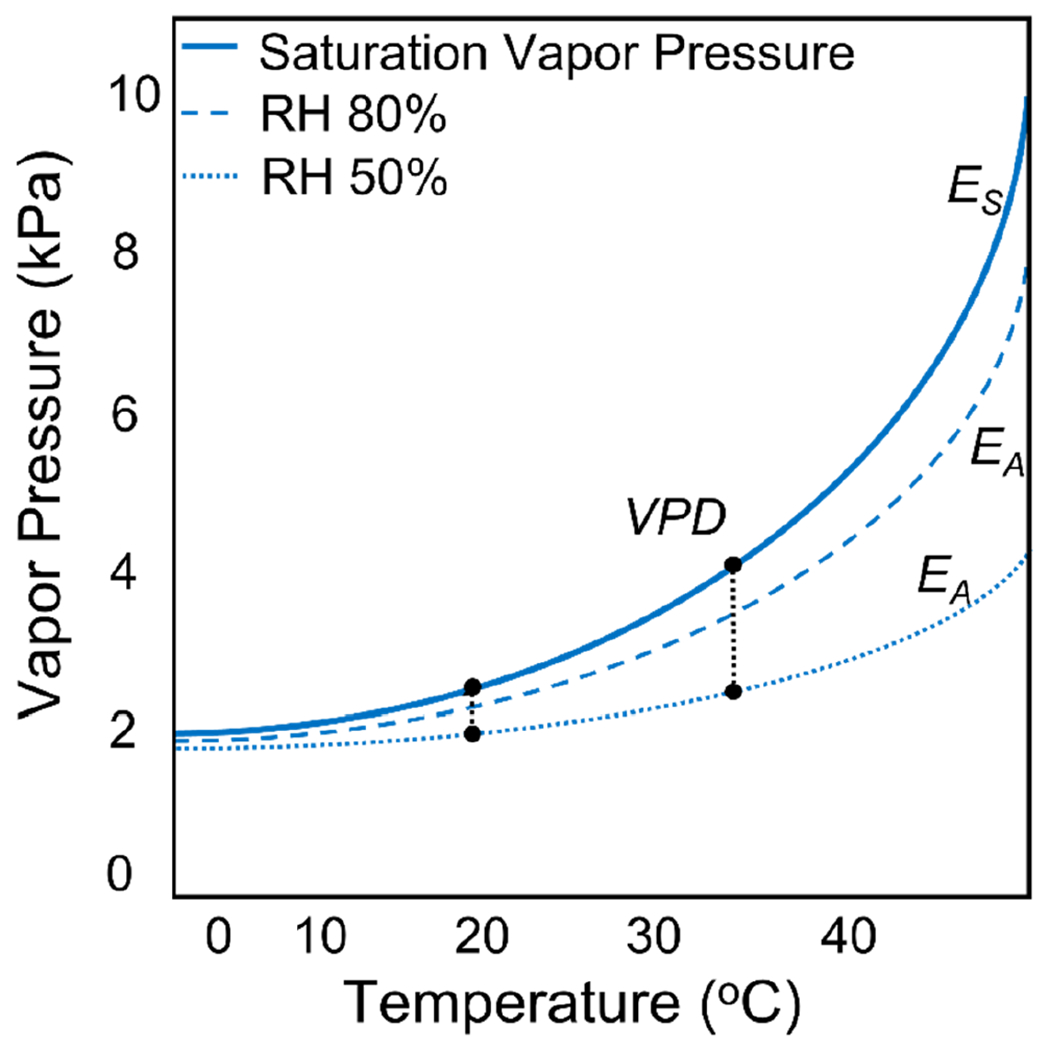
The total amount of water the air can hold, expressed here as saturation vapour pressure (*Es*), increases exponentially with temperature and is estimated as a function of temperature using the Tetens equation. The actual amount of water in the air, expressed here as vapour pressure (*Ea*), can be derived from relative humidity (*RH*) as *Ea* = *RH/*100 * *Es*. The vapour pressure deficit (*VPD*) is the absolute difference between *Es* and *Ea* and is an important metric of atmospheric moisture because it has a near linear relationship with evaporative potential. Thus, as temperature warms, for a given decrease in *RH*, there will be a larger increase in *VPD* and the amount of water stress mosquitoes experience.

The optimal regulation of both body temperature and water balance is crucial for organismal performance and fitness (Bradshaw, 2003). Due to the fundamental relationship that exists between temperature and the amount of moisture the air can hold (Figure 3), variations in both relative humidity and temperature will alter the degree of moisture stress ectothermic organisms, like mosquitoes, experience. For a given amount of atmospheric moisture, warmer temperatures result in higher saturation vapour pressures that reduce relative humidity and increase vapour pressure deficit (Figure 3). Depending on the ambient temperature, variation in relative humidity can exacerbate or buffer the negative effects of higher temperature on mosquito fitness and pathogen transmission. The current manner in which thermal performance of vector-borne pathogen transmission is conceptualized and empirically measured does not explicitly account for these effects. Even when relative humidity is held constant, increases in temperature will increase the vapour pressure deficit and the evaporative stress an adult mosquito experiences. Thus, it is currently unclear if the thermal maximum of a given trait, which is typically an upper lethal limit (Chown & Nicolson, 2004), is really being driven by temperature effects on metabolic function or rather is a function of dehydration and water stress on the organism. Understanding the physiological mechanisms underpinning mosquito responses to these abiotic constraints will be critical for predicting how transmission will shift with future anthropogenic change (Chown & Gaston, 2008; Deutsch et al., 2008; Dillon et al., 2010; Pörtner & Farrell, 2008).

**FIGURE 4:**
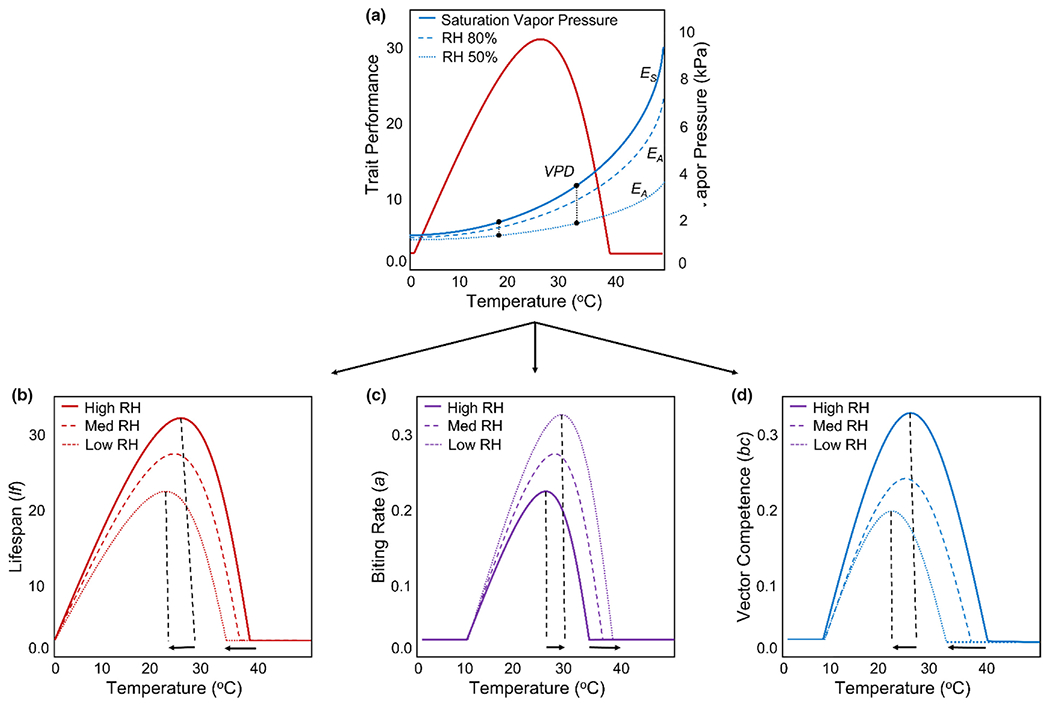
(a) Thermal performance is often measured by placing mosquitoes in different life stages and infection stages across a range of constant temperatures at a set relative humidity (typically between 70–90% RH). However, despite holding relative humidity constant, as temperatures warm there will be a corresponding increase in the vapour pressure deficit (*VPD*) and the amount of water stress mosquitoes experience. Overlaying these relationships (from Figure 1) on a given temperature-trait relationship demonstrates that the sensitivity of trait performance to variation in relative humidity should be highest on the descending limb of this relationship. *Es* = saturation vapour pressure, which increases exponentially with temperature and is estimated as a function of temperature using the Tetens equation. *Ea* = vapour pressure, meaning the actual amount of water in the air and can be derived from relative humidity (*RH*) as *Ea = RH*/100 * *Es.* (B–D) represent the hypothetical responses of three temperature-trait relationships to variation in relative humidity. These shifts are predicted to both decrease the thermal optimum and maximum for some traits (e.g., (b) lifespan and (d) vector competence) or increase them for others (e.g., (c) per capita biting rate).

**FIGURE 5:**
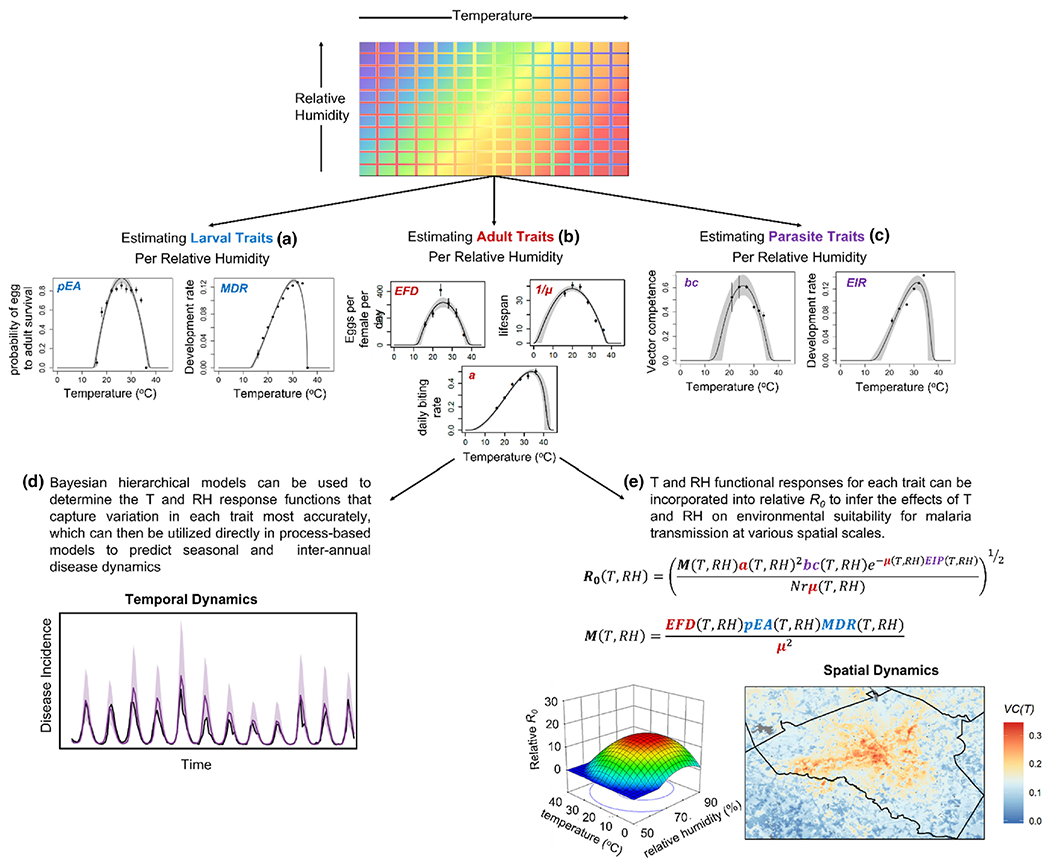
Laboratory work with field derived mosquitoes can be conducted to estimate the effect of multiple environmental variables on mosquito fitness, population dynamics and pathogen transmission. For example, mosquitoes could be housed across a range of constant temperature (*T*) and relative humidity (*RH*) conditions that are reflective of monthly field conditions. From these experiments, one can estimate the effects of variation in these environmental variables on key *larval traits* (a: mosquito development rate (*MDR*) and the probability of egg to adult survival (*pEA*)), *adult traits* (b: per capita mortality rate (*μ*), per capita eggs laid per day (*EFD*) and per capita daily biting rate (*a*)) and *parasite / pathogen traits* (c: vector competence (*bc*) and the extrinsic incubation period (*EIP)).* (d) Bayesian hierarchical models can be used to develop *T* and *RH* response surfaces for each trait, which can either be incorporated in process-based modelling approaches to infer effects on seasonal and interannual variation in vector-borne pathogen transmission dynamics. (e) Bayesian models can also be used to generate a *T* and *RH* dependent, relative *R*_*0*_ model that can be used to predict environmental suitability for pathogen transmission at various spatial scales. A crucial detail for modelling approaches, based on the evidence presented in Box 2, is that the effects of *T* and *RH* will be interactive, not additive. (Inset on temporal dynamics in (D) is from Santos-Vega et al. (2022) *Nature Communications;* doi: 10.1038/s41467-022-28,145-7. Figure is reproduced under Creative Commons Attribution 4.0 International Licence).

We utilize a trait-based approach that leverages a widely used relative *R*_*0*_ model (Mordecai et al., 2013, 2017, 2019; Murdock et al., 2017; Ryan, Lippi, & Zermoglio, 2020b; Shocket et al., 2020; Shocket, Ryan, & Mordecai, 2018a; Tesla et al., 2018; Villena et al., 2022; Wimberly et al., 2020) to present a framework that outlines the manner in which variation in relative humidity could influence the thermal performance of vector-borne pathogen transmission (Figures 4 and 5). Overall, we anticipate that variation in relative humidity could result in significant shifts in the qualitative shape of the temperature-trait relationship and cause these effects to vary with mosquito traits. Drawing from the literature on other ectotherms, insects and what little we do know for mosquitoes, we outline several hypotheses for how variation in relative humidity may affect the thermal performance of mosquito and pathogen traits (Table 2). We anticipate variation in relative humidity will be important throughout the mosquito life cycle, with the largest effects at temperatures that approach the upper thermal limit (*T*_max_) for a given trait, with little to no effect of variation in relative humidity on the predicted thermal minimum (*T*_min_) (Table 2). This hypothesis is based on the observation that for a given change in relative humidity, the corresponding change in vapour pressure deficit and evaporative stress will be greater at higher temperatures (Figures 3 and 4). How variation in relative humidity affects the predicted thermal optimum (*T*_opt_) of a given trait will be somewhat dependent on the specific trait as well as the magnitude of the effect at warmer temperatures.

The nature and magnitude of the effects of relative humidity and temperature variation on mosquito and pathogen traits important for transmission could differ depending on mosquito life stage. One way in which relative humidity and temperature interact to affect developing mosquitoes is through the evaporation rate of larval habitat, which is also determined by the size and surface area of the larval habitat and rate of water replenishment (Juliano & Stoffregen, 1994). A second type of interaction could involve altering some intrinsic factor of the larval environment such as surface tension, microbial growth or solute concentration (Juliano & Stoffregen, 1994; Pérez-Díaz et al., 2012). Causal evidence from semi-field experiments shows negative effects of high relative humidity at temperatures near or above the predicted thermal optimum for *Aedes albopictus* (Mordecai et al., 2017; Murdock et al., 2017) on larval survival and the probability of adult emergence (Murdock et al., 2017). One possibility is that both temperature and water vapour in the atmosphere will affect the surface tension of aquatic larval habitats. Warm temperatures and high humidity may cause larval habitats to have too little surface tension, while cool and dry larval environments may have too high surface tension (Pérez-Díaz et al., 2012; Singh & Micks, 1957), negatively affecting the ability of larval mosquitoes to breath, access nutrients and emerge from the pupal stage. In all likelihood, both types of effect could be important in the field. Thus, the effects of relative humidity on the rate of evaporation relative to larval development or shifts in intrinsic conditions of larval habitats could have substantial effects on the thermal performance curves for both mosquito development rate (*MDR*), the probability of egg to adult survival (*pEA*) and consequently the intrinsic growth rate of mosquito populations.

Once adults emerge from the larval environment, variation in relative humidity could potentially increase or decrease the predicted upper thermal limit for adult traits that are critical for mosquito population dynamics and transmission (Figure 4, Table 2). For example, decreases in relative humidity at warm temperatures could decrease mosquito survival (by increasing the per capita daily mortality rate (*μ*)) via increasing desiccation stress (Gaaboub et al., 1971; Lyons et al., 2014; Mayne, 1930). This, in turn, will decrease the temperatures at which mosquitoes can survive to become infected and to transmit vector-borne pathogens. Evidence from other insect systems (Chown and Nicolson (2004); Edney and Barrass (1962); Shelford (1918); Yu et al. (2010)) would predict that decreases in relative humidity at warm temperatures could also decrease the per capita daily biting rate (*a*) and production of eggs (*EFD*) by altering mosquito activity and blood feeding due to shifts in behaviour (e.g., utilization of specific habitats, times of day or times of season; Canyon et al. (1999); Drakou et al. (2020); Dow and Gerrish (1970); Gaaboub et al. (1971); Provost (1973)) and physiological responses (e.g., decreased metabolic rate) to increase desiccation resistance or tolerance (Chown & Davis, 2003; Marron et al., 2003). However, the evidence that does exist for mosquitoes suggests decreases in relative humidity can actually increase biting rates on hosts (e.g., *Culex pipiens, Ae. aegypti, An. quadramaculatus*; Hagan et al. (2018)). It remains unclear if this pattern would persist in the field for mosquito species that utilize sugar sources for hydration and nutrition, because nectar-feeding mosquitoes can increase sugar feeding behaviour when environmental conditions are dry (Fikrig et al., 2020).

Finally, we also anticipate that the development of mosquito-borne pathogens and parasites, and potentially mosquito susceptibility to infection, should be affected by variation in relative humidity under different ambient temperature conditions based on physiological acclimation responses (Beitz, 2006; Liu et al., 2016). Aquaporin water channels allow organisms to rapidly move water (aquaporins) or water and glycerol (aquaglyceroporins) across cellular membranes to promote cellular function. Mosquitoes utilize aquaporins and aquaglyceroporins to minimize water loss in desiccating environments (Liu et al., 2011) and to maintain glycerol concentrations to stabilize proteins when mosquitoes are exposed to high heat (Deocaris et al., 2006; Diamant et al., 2001; Liu et al., 2016; Tatzel et al., 1996). The physiological responses of mosquitoes to optimally thermo- and hydroregulate under sub-optimal temperature and relative humidity environments could also have consequences for the energy available to developing pathogen (Liu et al., 2016).

## IMPLICATIONS FOR UNDERSTANDING PATHOGEN TRANSMISSION AND CONTROL IN A CHANGING WORLD

Understanding the respective effects of variation in temperature and humidity, as well as any interaction between variables, will be critical for addressing how the regional and global distributions of mosquito vectors, and the seasonality and intensity of vector-borne pathogen transmission, will shift in response to future climate and land-use change. Based on the importance of maintaining optimal temperature and water balance in other organisms, we also argue that variation in temperature, humidity and water availability are important selective determinants driving local adaptation of mosquitoes to various environments as well as their capacity to respond to future environmental change. Finally, variation in temperature and humidity will also likely affect the efficacy, coverage and cost of disease control programs.

### Human-mediated environmental change

Human-mediated climate change is resulting in widespread and uneven changes in global temperature, humidity and precipitation patterns and more frequent extreme weather events (IPCC, 2021). In addition to climate warming, regional changes in humidity and precipitation will result in increased drought in some areas, while others become wetter (Konapala et al., 2020). If mosquitoes and their transmission cycles are more sensitive to humidity at higher temperatures, then future increases in wet vs. dry heat may have very different implications for mosquito populations and pathogen risk. Regional variation in temperature and relative humidity could have important implications for both the seasonal timing and peak of vector-borne disease (Santos-Vega et al., 2016, 2022) as well as pathogen persistence or emergence. For example, it has been suggested that future temperatures in tropical Africa will exceed the thermal optimum for malaria and result in reduced transmission (Mordecai et al., 2020). However, these tropical regions are characterized by humid heat, and malaria may persist if the maximum temperature for transmission increases at high humidity. Similarly, the potential for arboviruses to expand into warming temperate climates may be greater in regions with increasing humid heat vs. dry heat, which has not been considered in current mechanistic model projections of disease risk with various climate change scenarios [e.g., (Caldwell et al., 2021; Ryan, Carlson, et al., 2020a; Ryan, Lippi, & Zermoglio, 2020b)].

Land-use change is another key human driver affecting mosquito-borne disease transmission (Baeza et al., 2017). For example, urban landscapes are one of the most rapidly growing land cover types across the globe (United Nations, 2019), with the proportion of people living in urban environment projected to increase from 55% to 68% between now and 2050. High environmental heterogeneity in urban areas creates substantial variation in the local microclimates mosquitoes experience, through differences in temperature, moisture and wind speed (Stewart & Oke, 2012). These differences are mediated by the extent of impervious surfaces, the distribution of vegetation and the three-dimensional structure created by buildings and trees. Together, these changes result in urban heat and dry islands (Heaviside et al., 2017) with higher land surface (Yuan & Bauer, 2007) and near-surface air temperatures (Coseo & Larsen, 2014) and lower relative humidity (Hao et al., 2018; Heaviside et al., 2017; Lokoshchenko, 2017; Yang et al., 2017) compared to more vegetated landscapes. This fine-scale variation in mosquito microclimate can have significant implications for multiple mosquito species (e.g., *Aedes aegypti, Ae. albopictus, Anopheles stephensi*) that drive urban outbreaks of diseases (e.g., dengue, chikungunya, Zika and malaria) (Beebe et al., 2009; Heinisch et al., 2019; Li et al., 2014; Murdock et al., 2017; Stoddard et al., 2009; Takken & Lindsay, 2019; Thomas et al., 2016, 2017).

Small-scale variation in temperature and relative humidity could also have important implications for the spatial distribution of risk in urban environments (Figure 5). Recent studies that combine field experimentation with direct monitoring of urban microclimates and mosquito abundance demonstrate that fine-scale variation (e.g., individual neighbourhoods or city blocks) in both temperature and relative humidity can have important implications for mosquito life history, population dynamics and disease transmission within urban environments (Evans et al., 2019; Evans, Shiau, et al., 2018b; Murdock et al., 2017; Wimberly et al., 2020). Thus, neighbourhoods with a high proportion of impervious surfaces that experience mean temperatures near or exceeding the thermal optimum for transmission could experience even higher decreases in vectorial capacity than what models would predict from temperature alone, if drier conditions increase desiccation stress and reduce mosquito survival.

To generalize the effects of changing temperature and humidity across diverse locations and into the future, it will be necessary to develop a conceptual framework that incorporates the psychometrics of temperature and atmospheric moisture with mosquito biology and the natural and built environments in which transmission occurs. Incorporating the effects of humidity into hierarchical models and assessment of mosquito population dynamics and disease transmission will increase the precision of mapping environmental suitability, both globally and regionally with human-mediated environmental change, as well as across heterogeneous human-modified landscapes.

### Local adaptation and capacity to adapt in the future

There is growing interest in the factors driving adaptation of mosquitoes to local environmental conditions for providing insights into the long-term responses of mosquito species to future warming. Mosquito species are composed of an array of locally adapted populations across their respective ranges. Substantial genetic variation exists in mosquito species (Fouet et al., 2017; Holt et al., 2002; Kang et al., 2021; Maffey et al., 2020; Pless et al., 2020; Yurchenko et al., 2020) and at fine-spatial scales (Ayala et al., 2020; Carvajal et al., 2020; Gutiérrez et al., 2010; Jasper et al., 2019; Matowo et al., 2019), with significant consequences for transmission potential (Azar et al., 2017; Palmer et al., 2018; Vega-Rúa et al., 2020). This genetic variation can interact with local environmental conditions to impact the capacity of mosquito vectors to transmit human pathogens (e.g., dengue; Gloria-Soria et al. (2017) and chikungunya; Zouache et al. (2014)). Yet, we still do not have a clear understanding of what environmental factors are driving this differentiation.

The work that has been done in this area to date has largely focused on the effects of temperature variation in driving local adaptation of current mosquito populations (Couper et al., 2021; Sternberg & Thomas, 2014). However, research from the broader field of ectotherms [e.g., reviewed in Rozen-Rechels et al., 2019, vertebrates; Chown et al., 2011, insects] suggests that selection on thermal response curves are constrained by other metabolic stressors, like desiccation stress, as temperatures warm. For example, a study on 94 *Drosophila* species from diverse climates found substantial variation in the upper thermal limits among species. Further, the species specific CT_max_ correlated positively with increasing temperature in dry environments, with species from hot and dry environments exhibiting higher heat tolerance. However, this relationship completely disappeared for species inhabiting wet environments suggesting temperature as a selective force is less important when humidity is high (Kellermann et al., 2012). A similar study in ectothermic vertebrates (400 lizards), found the thermal optimum to be more strongly related to ambient precipitation than to average temperature (Clusella-Trullas et al., 2011). Environmental mean temperature was only found to be predictive of the lower thermal limit (CT_min_) (Clusella-Trullas et al., 2011).

Both common garden and experimental evolution studies, two standard approaches to measure local adaptation and evolutionary potential of a particular species, could be incorrectly attributing observed phenotypic responses to temperature selection when they could be responding to a combination of energetic effects and moisture stress. This impacts our ability to accurately characterize thermal response curves of mosquitoes, as well as their capacity to adapt to future environmental change. From our conceptual framework outlined above (Figure 5), we would predict that the current approach to studying local adaptation, steeped in metabolic theory of ecology, will be most predictive of mosquito population responses to future warming in regions of the world that currently exist below the species specific thermal optima (*T*_opt_). However, for mosquito populations that inhabit environments above their thermal optima, humidity will be an important determinant of their capacity to respond to future environmental change. For example, mosquito populations in warm and wet, humid environments may have less capacity to adapt to future climate change in a warming and drying environment than what would be predicted from evolutionary models that consider the effects of temperature alone. Conversely, mosquito populations that currently live in warm and dry environments may have a greater capacity to adapt to warming conditions if they exhibit higher heat tolerance than their counterparts inhabiting wetter areas of the geographic distribution.

### Controlling mosquito populations and disease transmission

There have been several mechanistic modelling efforts to understand how regional and seasonal environmental variation will impact the relative reproductive number of a pathogen, the intensity of human transmission and the efficacy of key disease interventions (e.g., Zika; Ngonghala et al. (2021), schistosomiasis; Nguyen et al. (2021)). These studies have, again, focused largely on the effects of ambient temperature. However, seasonal and regional variation in humidity and precipitation could extend or shorten the transmission season and magnify or depress the intensity of epidemics as predicted from models incorporating the effects of temperature alone (Huber et al., 2018; Ngonghala et al., 2021). For example, this is likely to be the case in seasonally dry environments where mosquito-borne disease transmission tends to be highest during or just after the rainy season and lowest during the hottest / driest parts of the season due to seasonal shifts in mosquito habitat, as well as the effects of temperature and humidity on mosquito and pathogen traits relevant for transmission.

How variation in humidity affects the efficacy of current and novel mosquito control interventions also needs to be considered. Many novel mosquito control technologies involve the mass release of males that have been sterilized or genetically engineered to pass on traits that confer either severe fitness costs (i.e., population suppression approaches; Alphey et al., 2010; Wilke & Marrelli, 2012; Wang et al., 2021) or enhanced resistance to human pathogens (i.e., population replacement approaches (Carballar-Lejarazú & James, 2017; Hegde & Hughes, 2017; Wilke & Marrelli, 2015)). For example, the *w*Mel strain of the symbiont *Wolbachia* can prevent dengue, chikungunya and Zika transmission in *Ae. aegypti* (Aliota, Peinado, et al., 2016a; Aliota, Walker, et al., 2016b; Moreira et al., 2009; Ye et al., 2015). Experimental work has determined that *w*Mel infections are temperature sensitive, with high temperatures causing reductions in *Wolbachia* density (Foo et al., 2019; Gu et al., 2022; Ross et al., 2017, 2019, 2020; Ulrich et al., 2016) and temperature variation affects the host-pathogen interaction and the outcome of infection in *Wolbachia*-infected mosquitoes (Murdock, Blanford, Hughes, et al., 2014a). Based on the relationship between temperature and water balance laid out in this paper, further experiments should examine whether *Wolbachia* infections are limited by temperature alone or by cellular water availability, and examine what role mosquito desiccation stress plays in limiting *Wolbachia* abundance within mosquitoes at varying temperature.

Furthermore, thermal performance differs between insecticide resistant vectors and their susceptible counterparts, with important implications for assessing fitness costs associated with insecticide resistance (Akinwande et al., 2021). Thus, insecticide resistant mosquitoes may have to optimize temperature and water needs across environmental constraints differently and, therefore, be affected by changes in humidity, with potentially important consequences for population dynamics, mosquito-pathogen interactions and transmission. Identifying these environmental constraints on efficacy and coverage will be critical for the successful implementation of current and future control programs (Parham & Hughes, 2015).

## CONCLUSIONS AND FUTURE DIRECTIONS

Sufficiently understanding the performance of insect vectors within the natural environmental mosaics where they occur will require substantially more data on the spatial and temporal complexities in microclimate, behavioural responses to temperature and humidity change, plasticity in thermal tolerance traits and the eco-physiological mechanisms of vector water balance, coupled with broader understanding of the general relationships between water and temperature described in this paper. We have collated these goals into a general framework incorporating humidity into research questions and temperature-dependent mechanistic models (Figure 5 & Box 2). We intend for the evidence and theory presented here to be signposts for future research, leading to a collective broadening in our understanding of insect vectors and how their responses to climate variables will affect parasite transmission.

## Figures and Tables

**TABLE 1:** Summary of the published literature that investigated the effects of relative humidity on mosquitoes, organized by life history trait, presented with a summary of the effect of RH.

Life history trait	Range explored (RH%)	Effect of RH	Mosquito species	References
Longevity/Survival/Desiccation tolerance	5–100	Increased RH significantly increased female longevity; tendency to survive longest at low temp—high humidity combinations and females to survive longer than males	*Anopheles gambiae, An. stephensi, An. subpictus, An. culicifacies, An. pharoensis, An. arabiensis, An.funestus, Ae. aegypti, Ae. albopictus, Ae. togoi, Ae. paullusi, Culex pipiens, Eretmapodites chrysogaster*	Alto et al. (2015), Bayoh (2001), Canyon et al. (2013), Costa et al. (2010), Gaaboub et al. (1971), Gray and Bradley (2005), Hylton (1969), Lega et al. (2017), Lewis (1933), Lomax (1968), Lyons et al. (2014), Mayne (1930), Mogi et al. (1996), Reiskind and Lounibos (2009) Schmidt et al. (2018) and Urbanski et al. (2010)
Egg production	34–95	Increased RH increased egg production; significantly lower numbers of eggs and delayed oviposition in dry conditions	*An. pharoensis, Ae. aegypti, Ae. taeniorhynchus, Ae. vexans, Ae. trivittatus*	Canyon et al. (1999, 2013), Costa et al. (2010), Gaaboub et al. (1971), Lega et al. (2017) and Mcgaughey and Knight (1967)
Activity/Behaviour	10–30, up to 100	Mosquito activity increases with increasing relative humidity to a point (~90%) and then precipitously declines, also has a general lower limit at ~40%.	*An. quadrimaculatus, An. earlei, An. walkeri, An. crucians, Ae. aegypti, Ae. sollicitans, Ae. cantator, Ae. vexans, Ae. trivittatus, Ae. canadensis, Ae. communis, Ae. sticticus, Ae. taeniorhynchus, Ae. vigilax, Cx. pipiens, Cx. fatigans, Cx. restuans, Cx. nigripalpus, Cx. sitiens, Coquillettidia perturbans, Psorophora confinnis, Uranotaenia sapphirina*	Bidlingmayer (1974, 1985), Dow and Gerrish (1970), Grimstad and DeFoliart (1975), Hagan et al. (2018), Johnson et al. (2020), Platt et al. (1957, 1958), Provost (1973), Rowley & Graham (1968), Rudolfs (1923, 1925), Thomson (1938), Witter et al. (2012) and Wright & Knight (1966)
*Plasmodium* infection	39–100	Mixed or unclear effects of humidity	*An. stephensi, An. subpictus, An. culicifacies, An. fuliginosus, Cx quinquefasciatus, Cx. fatigans*	Knowles and Basu (1943) and Mayne (1930)
Egg hatching	Real-world RH data; 0–100	Adding RH data to a predictive model focused on egg hatching made the model more accurate. Egg hatching worse at lower humidities	*Ae. aegypti*	Albernaz et al. (2009), Bar-Zeev (1957) and Lega et al. (2017)
Microclimate preference upon emergence	75, 86	Newly emerged adults with no access to water or sugar preferred cooler, more humid refugia.	*An. gambiae, An. stephensi, Ae. aegypti and Cx. pipiens*	Kessler and Guerin (2008)

**TABLE 2:** Predictions for the interactive effects of relative humidity & temperature on different mosquito traits.

Trait	Definition	*T*_min_	*T*_opt_	*T*_max_
*MDR*	Mosquito development rate (1/days)	No change	?	evaporation ↓ with ↑ RH = ↑ or no Δ in *T*_max_ no evaporation with ↑ RH = ↓*T*_max_
*pEA*	Probability of egg to adult survival	No change	?	evaporation ↓ with ↑ RH = ↑ *T*_max_ no evaporation with ↑ RH = ↓ *T*_max_
*EFD*	Per capita no. of eggs produced daily per female (1/days)	No change	?	RH ↑ = ↑ or ↓ *T*_max_
*a*	Per capita female biting rate (1/days)	No change	?	RH ↑ = ↑ or ↓ *T*_max_
*μ*	Per capita mosquito mortality rate (1/days)	No change	?	RH ↑ = ↑ *T*_max_
*bc*	Probability of becoming infectious	No change	?	RH ↑ =? *T*_max_
*EIR*	Extrinsic incubation rate (1/EIP or 1/days)	No change	?	RH ↑ = ↑ *T*_max_

## Data Availability

Data sharing not applicable to this article as no datasets were generated or analysed during the current study.

## Box 1 Different measurements of humidity.

In the most general sense, humidity is a measure of water vapour in the atmosphere, which is one important component defining an organism’s risk of desiccation. In practice, humidity can be quantified in multiple ways. A fundamental characteristic is the *vapour pressure*, which is defined in meteorology as the partial pressure of water vapour in the atmosphere. Vapour pressure affects the movement of atmospheric moisture, which diffuses from higher to lower vapour pressures (Allen et al., 1998; Wexler & Greenspan, 1971). The *saturation vapour pressure* is the maximum vapour pressure of a completely saturated parcel of air and is a non-linear increasing function of temperature (Figure 1). The *dew point temperature* is defined as the temperature to which air would need to be cooled for the saturation vapour pressure to equal the actual vapour pressure and is related to the total amount of moisture in the air. *Absolute humidity* is the mass of water vapour per unit volume of air, whereas *relative humidity* is the ratio of the actual water vapour pressure divided by saturation vapour pressure (at ambient temperature and pressure) (Lawrence, 2005). The *vapour pressure deficit* is the difference between saturation vapour pressure and the actual vapour pressure. Vapour pressure deficit is a key term in the numerator of the Penman-Monteith equation (used for calculating evaporation and evapotranspiration, see Allen et al., 1998; Chiew et al., 1995). Thus, if other environmental factors are constant, the rates of evaporative water loss from open water bodies and vegetated surfaces are expected to increase as a linear function of vapour pressure deficit.

Of these humidity measures, relative humidity is most commonly provided in meteorological datasets. Relative humidity is a unitless ratio in which the influence of temperature on the saturation vapour pressure has been removed, which has several important implications. Even if the total amount of water in the air is constant, relative humidity will vary with temperature. This effect is most commonly observed in the diurnal cycle of humidity, which reaches a minimum during the warmest time of day and a maximum at the coolest times of night. When comparing two observations with similar relative humidities but different temperatures, the observation with the higher temperature will have a higher vapour pressure, saturation vapour pressure and vapour pressure deficit. Thus, the potential for evaporative water loss at a given relative humidity is inherently sensitive to changes in temperature (Figure 1).

## Box 2 Future Questions

- **Direct effects of humidity**
  - To what extent will humidity affect parasite and pathogen development within mosquitoes, and what are the underlying mechanisms?
  - How does humidity affect thermal performance curves of both mosquitoes and pathogens? Does it impact the variation in the thermal optimum, limits, operative range or performance magnitude?
  - How do experimental conditions (e.g., constant vs. diurnally fluctuating environments, different humidity values used to assess thermal performance) influence the interpretation and variation in thermal performance, including in semi-field experiments?
  - Does humidity affect different mosquito populations and species similarly?
  - How do background humidity conditions influence the interpretation and variation in thermal performance characterized in laboratory experiments or in semi-field experiments?
  - How much of the decrease in thermal performance at thermal extremes (CT_min_, CT_max_) can be explained by mosquito water balance, rather than temperature alone?
  - Does the environmental niche, defined by temperature and water constraints, explain current distributions and abundances of key mosquito vectors and vector-borne diseases?
  - At what spatial or temporal scales should measurement, inference and prediction be implemented for mosquito population dynamics, disease transmission and control, in the context of temperature and humidity? Do these scales differ between the two climate variables?
- **Consequences of interactive effects of temperature and humidity**
  - How do humidity and desiccation stress shape the evolution of thermal performance? Which traits and elements of thermal performance (e.g., thermal optimum, thermal limits, operative range) will experience, and respond to, selection?
  - How will changes in humidity resulting from future climate and land-use change affect the potential for mosquito thermal performance to adapt to warming temperatures?
  - Do differences in the ability to thermo-hydroregulate across mosquito species help explain evolutionary differences in life history and how these species utilize their environments (e.g., day, night or dawn/dusk host seeking; circadian rhythms in the production of malaria infectious stages in human hosts; propensity to sugar feed; microhabitat selection of resting mosquitoes; oviposition site selection; seasonal and diurnal timing of mating; duration of dry diapause or overwintering)
  - How will population genetic variation impact mosquito adaptability to local microclimates? And to what extent will phenotypic plasticity in traits affecting thermal tolerance and water balance influence persistence of mosquito populations, communities and species?
  - How will current and novel (e.g., *Wolbachia*) vector control strategies be impacted by variation in humidity and temperature? How will this affect the timing, distribution, duration and efficacy of a given intervention?
